# Fermented cottonseed and rapeseed meals outperform soybean meal in improving performance, rumen fermentation, and bacterial composition in Hu sheep

**DOI:** 10.3389/fmicb.2023.1119887

**Published:** 2023-03-16

**Authors:** Halidai Rehemujiang, Hassan Ali Yusuf, Tao Ma, QiYu Diao, Luxin Kong, Lingyun Kang, Yan Tu

**Affiliations:** ^1^Key Laboratory of Feed Biotechnology of Ministry of Agriculture and Rural Affairs, Institute of Feed Research of Chinese Academy of Agricultural Sciences, Beijing, China; ^2^Faculty of Veterinary Medicine and Animal Husbandry, Somali National University, Mogadishu, Somalia

**Keywords:** fermented total mixed ration, cottonseed meal, rapeseed meal, Hu sheep, rumen microbiota

## Abstract

**Background:**

This study examined the effects of substituting cottonseed meal (CSM) or rapeseed meal (RSM) for soybean meal (SBM) on Hu sheep performance, rumen fermentation, and bacterial composition. 51 four-month-old indigenous male Hu sheep with starting body weights of 22.51 ± 2.84 kg and similar origins were randomly assigned to three treatments; (1) non-fermented total mixed ration (TMR) with SBM (CK), (2) fermented TMR containing CSM (F-CSM group), and (3) fermented TMR containing RSM (F-RSM group).

**Results:**

The three groups’ intake of dry matter differed significantly (*p* < 0.05). In terms of average daily gain, the F-RSM group outperformed the CK and F-CSM groups (*p* < 0.05). The pH of the rumen was substantially lower in the CK group than in the F-CSM and F-RSM groups (*p* < 0.05), and the F-CSM group had greater amounts of volatile fatty acids (VFA) than the F-RSM and CK groups. In comparison to the CK group, the microbial crude protein yield was significantly higher in the F-CSM and F-RSM groups (*p* < 0.05). The F-CSM group significantly outperformed the F-RSM group of pepsin and cellulose enzyme activity (*p* < 0.05). The relative abundance of *Bacteroidetes* was greater in the CK and F-RSM groups compared to the F-CSM group (*p* < 0.05). In comparison to the other groups, *Firmicutes* were less abundant in the CK group (*p* < 0.05). *Prevotella* was present in a higher relative abundance in the F-CSM and F-RSM groups than in the CK group (*p* < 0.05). *Prevotella* was greater in relative abundance in the F-CSM and F-RSM groups than in the CK group (*p* < 0.05). The relative abundances of *Veillonellaceae_UCG-001* and *Lachnospiraceae_XPB1014* correlated with rumen butyric acid content and NH_3_-N content (*p* < 0.05). Gene function prediction revealed that replacing SBM with F-CSM or F-RSM in the diet of Hu sheep can promote glycan biosynthesis and metabolism.

**Conclusion:**

The replacement of F-CSM and F-RSM for SBM has an influence on the richness and diversity of rumen bacteria at the phylum and genus levels. Replacement of SBM with F-CSM increased VFA yield and further promoted the performance of Hu sheep.

## 1. Introduction

The worldwide population is forecast to spread 9.7 billion (Olimid and Olimid, 2019), while universal need for meat and milk is expected to grow by 57 and 48%, respectively (Alexandratos and Bruinsma, 2012). As an outcome, it is estimated that livestock production will rise by 21% between 2010 and 2025 (Mottet et al., 2017). The universal need for livestock protein for human nourishment is increasing exponentially, increasing the price of animal feed concentrates (Tufarelli et al., 2018). To overcome the problem, it is vital to adopt different sources of feed and strategies using of alternative feeding methods. By-products are the sole viable option for making low-cost feed ingredients; they are generated during food processing and production and thus are not appropriate for human consumption (Xu et al., 2007). In ruminant diets, CSM and RSM may be appropriate alternatives to SBM as they are less expensive and relatively more widely accessible regionally (Zeng, 2020).

China has a large supply of CSM and has long been measured as a reasonable alternative to SBM (Wang et al., 2017). As a by-product of cottonseed oil manufacturing, CSM exhibits a crude protein content of 34–40%, crude fibre content of 11%, vitamin B, and organic phosphorus, among other nutrients (Zhang et al., 2018). However, due of the presence of Free gossypol (FG), a poisonous that may significantly influence animal development and reproductive and digestive development, as well as cause anomalies in internal organs, the use of CSM in animal diets is restricted (Francis et al., 2001; Robinson et al., 2001). Cotton-rich feed is a common source of protein for ruminants, particularly in cotton-growing regions such as India, China, and the United States, where it is used to replace soybean meal (SBM) (Heuze et al., 2015). However, CSM is now a cost-effective source of high protein (Nie et al., 2015). RSM, a by-product of the rapeseed oil industry, has a high protein concentration and a well-balanced amino acid composition (Wanasundara et al., 2017; Ashayerizadeh et al., 2018), making RSM appropriate for use in livestock feed. Nonetheless, because of the presence of glucosinolates (Huisman and Tolman, 1992; Tripathi and Mishra, 2007) and other anti-nutritional factors, RSM can poison animals and impair their development (Nagalakshmi et al., 2003). As a result, there is a limited usage of CSM and RSM in livestock diets.

Several approaches had been used to decrease the anti-nutritiona factors of CSM, such as calcium hydroxide (Nagalakshmi et al., 2003), chemical treatment with ferrous sulfate (Barrett et al., 1997), and microbial fermentation (Weng and Sun, 2006). To reduce the anti-nutritional factors of RSM, methods such as inactivation of myrosinase, solvent extraction, steam removal, and liquidation have been applied. Still, such methods also have some disadvantages including loss of protein, high expense, commercial relative unimportance, and environmental pollution (Pal and Walia, 2001). Among the different methods, microbial fermentation can help in increasing crude protein and other nutritional content, reduce toxin content, and maintain stomach microbial stability of the plants (McSweeney et al., 2001; Xu et al., 2020). Furthermore, our preliminary study showed that the nutritious value of CSM and RSM can be increased by microbial fermentation, while the anti-nutritional factors (FG and glucosinolate) in them can be reduced.

The diverse and adaptable rumen microbiota, which includes bacteria, archaea, fungus, protozoa, and viruses, allows for the utilization of a variety of food materials, including recalcitrant plant wall elements, and the production of assimilable energy and nutrients needed by ruminants (Kolver et al., 2001). Undoubtedly, the rumen microbiome also allows for nutritional manipulations. In fact, functions of the rumen microbiota and a number of the production phenotypes of ruminants are linked (Zhang et al., 2017; Li et al., 2019; Zhang Y. et al., 2020). VFAs, which supply 70% of the required energy, can be produced by ruminants from solid diet. The microbes in the rumen enable this (Anantasook et al., 2013; Khan et al., 2016). The variations in feed efficiency among individual cattle can be primarily attributed to rumen bacteria and VFAs (Liang et al., 2017; Zhou et al., 2018; Zhang Y. X. et al., 2020). In a study by Zhang et al. (2018), the amount of FG and total gossypol in fermented CSM fell by 78 and 49%, respectively, under the best fermentation conditions in comparison to the control. These results indicate that rumen bacteria have considerable capacity to digest gossypol, which helps to explain why ruminants have a high tolerance for gossypol (Tang et al., 2018). However, the microbes involved in such rumen fermentation are not known. Moreover, the impact of CSM and RSM on rumen fermentation and rumen microorganisms has only been studied by a few researchers. Therefore, this study’s objective was to determine whether replacement of SBM with F-CSM or F-RSM affected growth performance, rumen fermentation and bacterial composition in Hu sheep.

## 2. Methods and materials

### 2.1. Ethics committee approval

The experiment was conducted in compliance with the Guidelines for Experimental Animals (2006) established by the Ministry of Science and Technology (Beijing, China), and it was given the go-ahead by the Chinese Academy of Agricultural Sciences’ Animal Ethics Committee (AEC-CAAS-20190517).

### 2.2. Animals, diets, and experimental design

51 four-month-old native male Hu sheep with ancestries that were identical and an initial weight of 22.51 ± 2.84 kg were acquired from China’s Inner Mongolia autonomous region. The Hu sheep were divided into three groups (17 Hu sheep each) using a single-factor randomized block design: CK group, F-CSM group, and F-RSM group. Subsequently, they were fed the total mixed ration (TMR). A stall (3 m × 1.5 m) equipped with individual feed and water buckets was utilized to maintain the Hu sheep. Unrestricted access to TMR and clean water was given to the Hu sheep.

Along with SBM, CSM, or RSM meals, wheat bran, maize, and corn stalks were used in the TMR formulation. Additionally, urea, premixed vitamins, and fat powder were added. The dietary feed was formulated according to the guidelines for meeting the nutrient requirements of sheep and allowing them to gain 300 g per day (NRC, 2007). Table 1 is a list of the TMRs’ components and chemical composition. Based on our previous studies (Yusuf et al., 2021), F-CSM was prepared with a 50% moisture content and a 1:5 mixture of microbial strains (1.0 × 10^9^ CFU/kg DM (dry matter) of *Bacillus clausii* and 5.0 × 10^9^ CFU/kg DM of *Saccharomyces cariocanus*). F-RSM was also prepared with a 50% moisture content and a mixture of microbial strains (1.0 × 10^10^ CFU/kg DM of *B. clausii* and 5.0 × 10^10^ CFU/kg DM of *S. cariocanus*). In a 500 kg fermenter machine, the mixture was fermented (Model SSJX-WH-3.0; Shengshun Machinery Manufacturing Co., Ltd., Shenyang, China) for 60 h at 32°C (F-CSM group) or 28°C (F-RSM group). Following fermentation, the mixture was uniformly mixed with whole corn silage before being bagged in plastic. Every 3 days, TMR (CK group) without fermentation was mixed.

**Table 1:** Ingredients and chemical composition of the TMRs.

	Groups^1^		
Items	CK^1^	F-CSM	F-RSM
Ingredients (% of DM)			
Corn	34	33.55	33.48
Wheat bran	12	12	12
Soybean meal	10	0	0
Cottonseed meal	0	10	0
Rapeseed meal	0	0	10
Fat powder	0	0.3	0.3
Urea	0	0.15	0.22
Whole corn silage	20	20	20
Corn stalk	20	20	20
Premix^2^	4	4	4
Total	100	100	100
Chemical compositions (% of DM)^3^			
Dry matter (fresh basis)	55.85	56.86	56.9
Crude protein	13.98	14.66	13.82
Eether extract	3.07	3.43	3.53
Neutral detergent fiber	27.66	26.15	27.74
Acid detergent fiber	18.04	17.28	15.74
Ash	7.99	8.07	8.65
Calcium	1.07	1.11	1.15
Phosphours	0.46	0.52	0.46

^1^CK, total mixed ration with soyabean meal; FTMR-CSM, fermented total mixed ration with cottonseed meal; FTMR-RSM, fermented total mixed ration with rapeseed meal. ^2^Premix provide following per kg to the total mixed rations: Vitamins A, 10000 IU; Vitamin D3,2,500 IU; Vitamin E,50 IU; Fe, 90 mg; Cu, 12.5 mg; Mn, 30 mg; Zn, 100 mg; Se, 0.3 mg; I, 1.0 mg; Co, 0.5 mg. ^3^All the value are measured.

During July to October of 2021, the experiment was conducted at the Chinese Academy of Agricultural Sciences’ Nankou Experimental Pilot Test Base (China’s Beijing). The study lasted for 97 days, including 10 days for adaptation, 80 days for the feeding trial, and 7 days for the apparent digestion trial. At 07:00 and 17:00 each day, *ad libitum* feedings were given, with the delivered amount adjusted considering a nearly 10% refusal rate.

### 2.3. Sample collection and analyze

#### 2.3.1. Growth performance

During all feeding periods, the diets and ort samples of individual Hu sheep were harvested daily to calculate the nutrient intake. The initial and final body weights were measured before the morning feed on the start and finish days. Afterwards, the dry matter intake (DMI), average daily gain (ADG) and feed conversion ratio (FCR) were calculated (Hassan et al., 2022a).

#### 2.3.2. Rumen fermentation and enzyme activity

At the end of the experiment, 2 h after the morning feeding, using an oral stomach tube, rumen fluid was taken (Anscitech Co., Wuhan, Hubei, China). A sample of rumen fluid (1.0 ml) and 0.3 ml of 25% metaphosphoric acid were mixed and stored at-20°C.till the VFA, microbial crude protein (MCP) and ammonia nitrogen (NH_3_-N) concentrations were investigated. The pH of rumen fluid was quickly determined using a portable pH detector (Testo-206-pH, Testo Co., Germany). The VFA concentration was determined using an Agilent Technology-78-90A gas chromatograph (Agilent Technologies, Waldbronn, Germany) coupled to an attached silica vessel column (30 mm × 0.25 mm × 0.25 μm film thickness; SP-3420A; Beifenrili Analyzer Associates, Beijing, China). A modified colorimetric technique was used to measure the NH_3_-N content (Chaney and Marbach, 1962). The microbial protein component of the rumen fluid was identified using an updated colourimetric technique at 595 nm (Verdouw et al., 1978). Utilizing materials acquired from the Chinese company Nanjing Jiancheng Institute of Biological Engineering Co., we determined the activities of amylase (CNPG_3_ method), pepsin (A080-1), and cellulase (A) in rumen fluid. The main instruments used were an HWS-12 water bath (Shanghai Blue Pard Instruments Co., Ltd.), a 721 spectrophotometer, a thermostat, and a PL-6906 microplate meter (Shanghai Youke Instrument Co., Ltd).

#### 2.3.3. DNA extraction and 16S rRNA pyrosequencing

The V3-V4 region of the 16S rDNA was amplified using specific primers with a barcode; the primer sequences were 338F: ACTCCTACGGGAGGCAGCAG; and 806R: GGACTACHVGGGTWTCTAAT. DNA was extracted using the kit (Omega Biotek, Norcross, GA, United States). By using 1% agarose gel electrophoresis to identify PCR products, they were then purified using the Agencourt AMPure XP nucleic acid purification kit. Equal quantities of the purified amplified products were mixed, and the “Y” shaped connection was connected. Magnetic bead screening was used to remove the connector’s self-connecting parts. The library template was enriched by PCR amplification to generate single-stranded DNA fragments, and MiSeq was used to construct and sequence the library.

#### 2.3.4. Sequence analysis

As stated previously, sequencing data was analyzed using the Quantitative Insights into Microbial Ecology (QIIME, v1.8.0) pipeline (Caporaso et al., 2010). In brief, raw sequencing reads that exactly matched the barcodes on the samples tested were assigned to them and identified as valid sequences. Low-quality sequences were detected by the following parameters: length of 150 bp, average Phred scores of 20, ambiguous bases, and > 8 bp of mononucleotide repetitions. FLASH was used to assemble paired-end reads (Magoc and Salzberg, 2011). Following chimera identification, UCLUST was used to group the remaining high-quality sequences into operational taxonomic units (OTUs) with 97% sequence identity. Using the default parameters, we selected a representative sample sequence from each OTU. By comparing the representative sequence set to the Greengenes Database and selecting the best hit, BLAST was used to classify OTUs. Each OTU’s abundance and taxonomy were then recorded in an OTU table for each sample. OTUs with less than 0.001% sequence content across all samples were discarded. A typical, rounded rarefied OTU table to even out sample-to-sample sequencing depth differences. We calculated richness estimates and diversity indices using the QIIME v1.8, including Shannon, Simpson, Chao 1, Observed species, Goods coverage, and PD entire tree. We obtained the species classification information corresponding to each OTU by annotating specific information for the communities at various levels and comparing OTU representative sequences using BLAST (2.6.0+). We used linear discriminant analysis (LDA) effect size analysis to identify species with significant differences in abundance among groups (Langille et al., 2013). TThe first method for identifying species with statistically significant differences in abundance among groups was the analysis of variance (ANOVA) test. The Wilcoxon rank-sum method was then used to investigate group differences. For the purpose of data reduction and an investigation of the significance of significant phenotypic differences between species, the algebraic linear discriminant analysis score was set to 3.0. To compare all samples, we used Principal Coordinates Analysis (PCoA) based on weighted UniFrac distances, and a distance-based matrices analysis was performed to identify sample differences.

#### 2.3.5. Microbial function prediction

To estimate the functions of the rumen microbial community, we used the Phylogenetic Investigation of Communities by Reconstruction of Unobserved States (PICRUSt v2.0.0^1^). The abundance of each functional category was determined using data on the functional orthologues, pathways, and enzyme commission numbers collected in accordance with the analysis module of the Kyoto Encyclopedia of Genes and Genomes (Langille et al., 2013).

#### 2.3.6. Statistical analysis

All data were represented using the mean standard deviation. A randomized complete block design with repeated measurements was utilized to analyze the data on rumen fermentation parameters. The Kruskal-Wallis technique in the R program was used to analyze the differences in alpha diversity, relative abundance at the phylum, family, and genus levels, and microbiota function among the three groups (v 4.0.3). The “ape” and “ggplot2” packages in R (v 4.0.3) were used to display PCoA, Venn, column, and linear discriminant analysis effect size (LEfSe) data, respectively. Using R (v 4.0.3), package “corrplot,” we investigated the relationship between the top 20 genera in all data (based on Spearman’s coefficient). The “igraph” package of the R (v 4.0.3) language was used to show the network of the top 20 genera. At P 0.05, all results related to intergroup differences were statistically significant.

## 3. Results

### 3.1. Effect on feed intake and performance

The Hu sheep in the CK, F-CSM, and F-RSM groups exhibited ADG of 192.10 g/d, 210.12 g/d, and 226.03 g/d, respectively; Compared to the CK group, Hu sheep ADG in F-RSM group significantly higher than CK and F-CSM group (*p* < 0.05). The F-CSM group had significantly higher DMI than the F-RSM and CK groups (*p* < 0.05). Additionally, the difference between both the FCR in the CK and F-CSM groups and the F-RSM group was statistically significant (*p* < 0.05; Table 2).

**Table 2:** Effects of the diets on growth performance of Hu sheep.

Items	Groups			SEM	*p* value
	CK^1^	F-CSM	F-RSM		
Initial weight (kg)	22.7	22.3	22.4	0.40	0.83
Final weight (kg)	39.7^b^	42.0^ab^	43.2^a^	0.67	0.40
Average daily gain (g/d)	192.1^b^	210.1^ab^	226.0^a^	0.03	0.03
Dry matter intake (g/d)	970.1^c^	1140.0^a^	1110.1^b^	0.04	<0.01
Feed conversion rate, feed/gain	5.4^a^	5.1^a^	4.9^b^	0.08	0.02

^1^CK, total mixed ration with soyabean meal; F-CSM, fermented total mixed ration with cottonseed meal; F-RSM, Fermented total mixed ration with rapeseed meal. ^a,b,c^Values with different superscripts within the same row differ (*p* < 0.05).

### 3.2. Effect on rumen fermentation

In comparison to the CK group, the pH of the rumen fluid was considerably lower in the F-CSM and F-RSM groups (*p* < 0.05; Table 3). Total VFA, acetate, propionate, and butyrate concentrations differed (*p* < 0.05) among the groups, with the F-CSM group having higher concentrations than the F-RSM and CK groups. However, the F-CSM group exhibited a higher proportion of propionate than that exhibited by the CK and F-RSM groups (*p* < 0.05). When compared to the CK group, the F-CSM and F-RSM groups had substantially greater NH_3_-N contents and MCP concentrations, respectively (*p* < 0.05).

**Table 3:** Effects of the diets on ruminal fermentation parameters of Hu sheep.

Items	Groups			SEM	*p* value
	CK^1^	F-CSM	F-RSM		
pH	6.4^a^	5.9^b^	6.0^b^	0.08	0.03
Total VFA (mmol/L)	79.6^b^	116.9^a^	62.9^b^	6.10	0.01
Acetate (%)	70.5^b^	70.7^b^	71.8^a^	4.34	0.01
Propionate (%)	15.9^b^	18.3^a^	16.5^b^	1.35	0.01
Butyrate (%)	13.6^ab^	11.0^a^	11.7^b^	0.82	0.02
A/P	4.4^a^	3.9^b^	4.3^a^	0.22	0.02
NH_3_-N (mmol/L)	4.9^b^	7.1^a^	7.1^a^	0.5	0.05
MCP (mg/100 ml)	3.1^b^	4.7^ab^	4.8^a^	0.4	0.04

VFA: volatile fatty acid; NH_3_-N: Ammonia nitrogen; MCP: microbial crude protein; A/P: a ratio of acetate to propionate. ^1^CK, total mixed ration with soyabean meal; F-CSM, fermented total mixed ration with cottonseed meal; F-RSM, fermented total mixed ration with rapeseed meal. ^a,b,c^Values with different superscripts within the same row differ (*p* < 0.05).

### 3.3. Effect on enzyme activity

The enzyme activities in each group are shown in Table 4. Adding different proteins to the TMR did not affect rumen α-amylase activity (*p* > 0.05). Pepsin and cellulose activities in the F-CSM group were substantially greater than those in the F-RSM group (*p* < 0.05). There was no significant difference between the CK and F-CSM groups (*p* > 0.05).

**Table 4:** Effects of the diets on ruminal enzyme activity of Hu sheep.

Items	Groups			SEM	*p* value
	CK^1^	F-CSM	F-RSM		
Alpha-Amylase(U/g)	38.6	40.8	35.3	0.97	0.06
Pepsin (U/mg)	14.4^ab^	15.5^a^	13.2^b^	0.39	0.04
Cellulose (U/mg)	2.3^ab^	2.6^a^	1.9^b^	0.12	0.04

^1^CK, total mixed ration with soyabean meal; F-CSM, fermented total mixed ration with cottonseed meal; F-RSM, Fermented total mixed ration with rapeseed meal. ^a,b,c^Values with different superscripts within the same row differ (*p* < 0.05).

### 3.4. Effect on the composition of microbial community OTU

**Figure 1:**
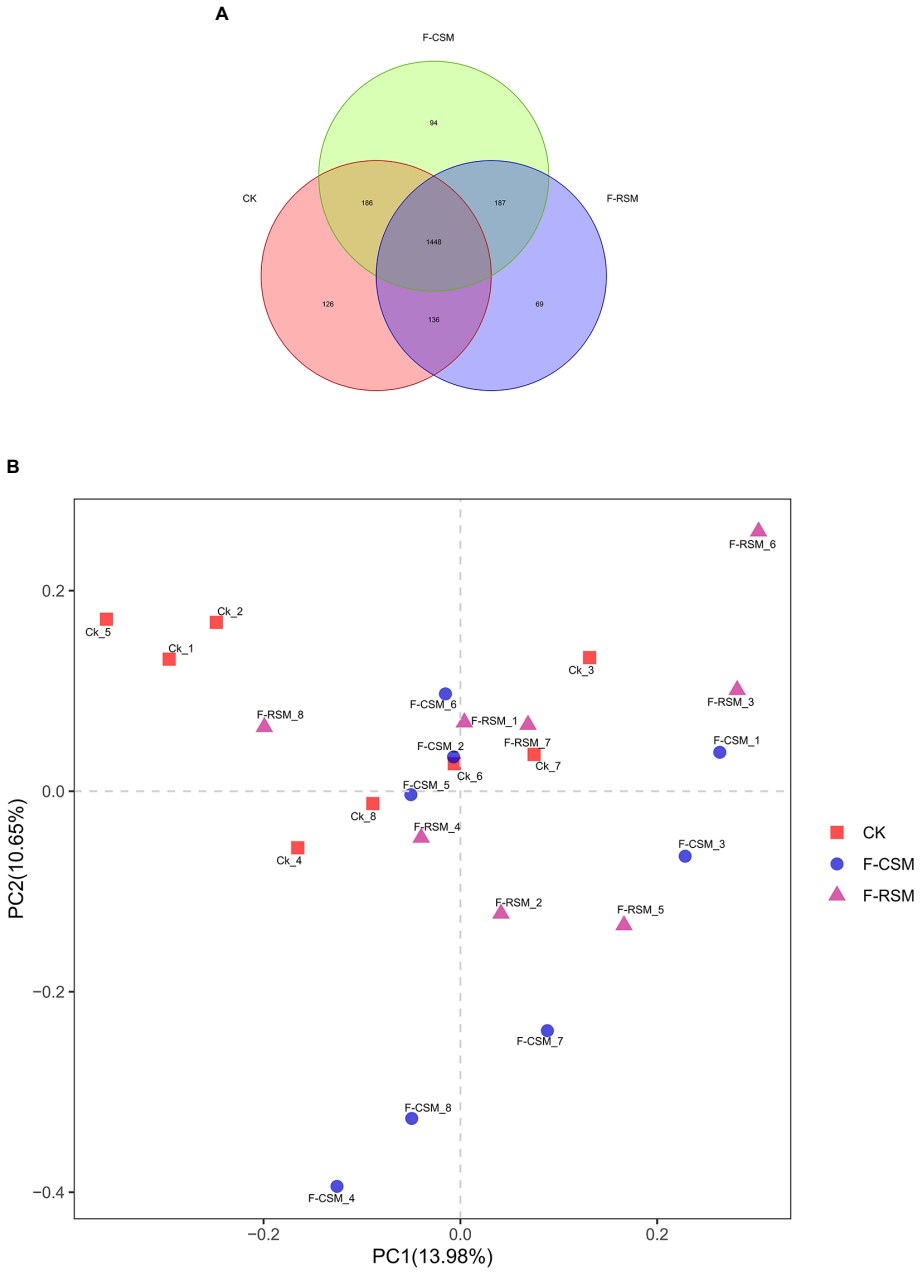
Effects of the diets on rumen microbiota of Hu sheep **(A)** Composition of rumen microbiota from Hu sheep fed three diets (OTU-level analysis). **(B)** PCoA analysis of rumen microbiota from Hu sheep fed different diets (%); CK, non-fermented group; F-CSM, fermented cotton seed meal TMR; F-RSM, fermented rapeseed meal TMR.

The OUT rank curve graphic gradually stabilized, indicating that the test samples’ high coverage (Figure 1). A total of 1,831,937 high-quality reads were obtained, for a sample size of 76,330 reads, composed the rumen microbiota. Nucleotide sequence identification (97%) resulted in a total of 2,285 OTUs (Figure 1A). Among these, 1,448 OTUs (63.37%) were shared among samples from different groups. There were no dietary effects observed, and PCoA analysis based on Weighted UniFrac or Bray–Curtis dissimilarity revealed that the rumen liquid fraction was not completely differentiated from other liquids (Figure 1B).

### 3.5. Effect on ruminal bacterial alpha diversity

**Figure 2:**
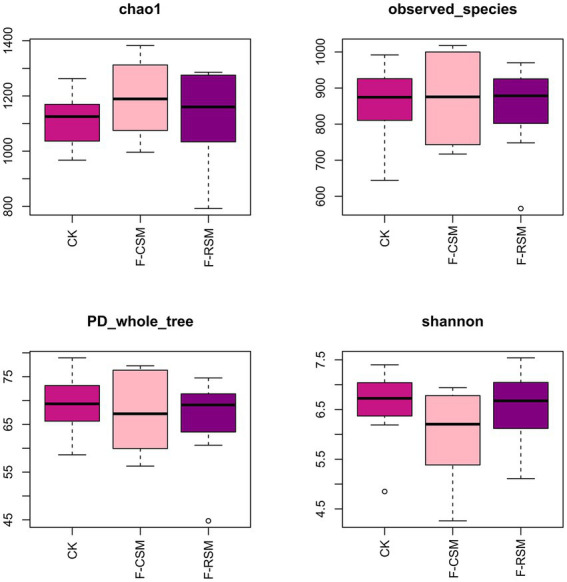
Effects of the diets on rumen alpha diversity of Hu sheep. CK, non-fermented group; F-CSM, fermented cotton seed meal TMR; F-RSM, fermented rapeseed meal TMR.

Analysis of alpha diversity (Figure 2) showed that the F-CSM group had numerically higher Shannon and community richness (Chao) indices than those in the CK and F-RSM groups; however, the differences were not significantly different (*p* > 0.05). The detected species were not substantially different among groups (*p* > 0.05). The Simpson index showed no discernible differences between the three groups (*p* > 0.05).

### 3.6. Effect on ruminal bacteria at phylum and genus levels

**Figure 3:**
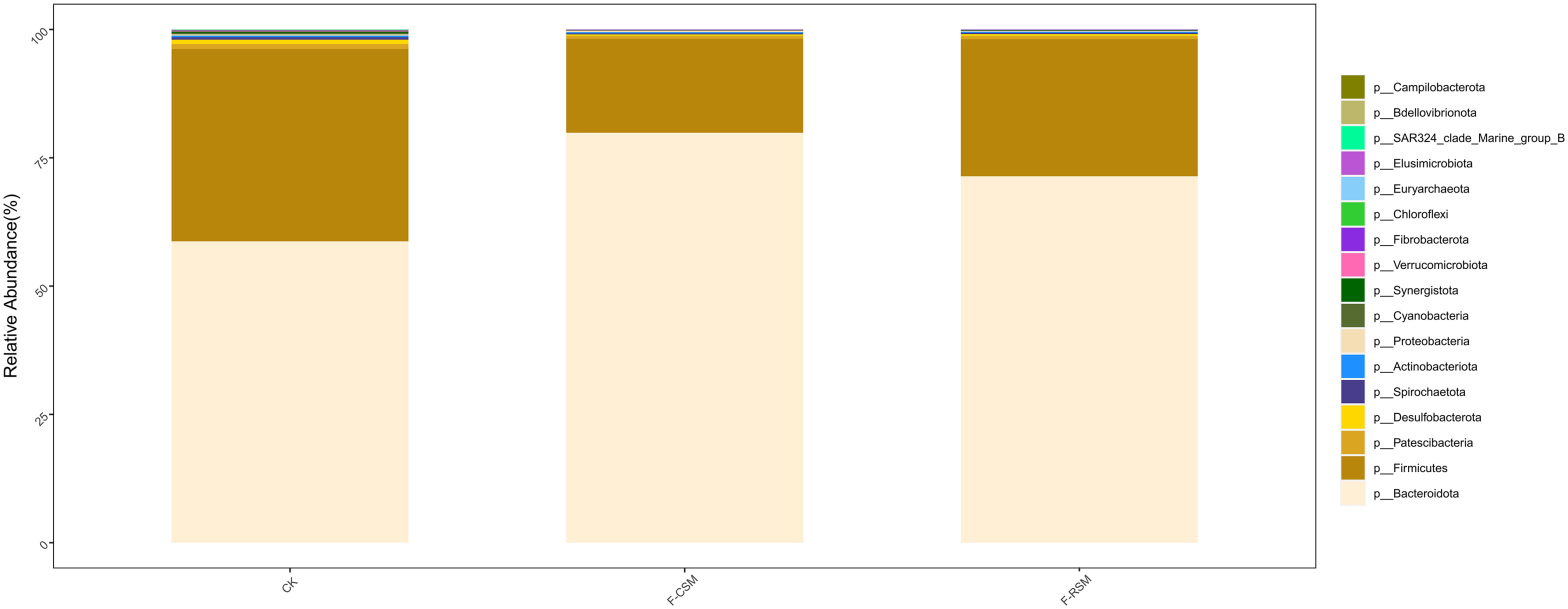
Effects of the diets on compositional profifiles of rumen microbiota of Hu sheep (Phylum level; %). CK, non-fermented group; F-CSM, fermented cotton seed meal TMR; F-RSM, fermented rapeseed meal TMR.

**Figure 4:**
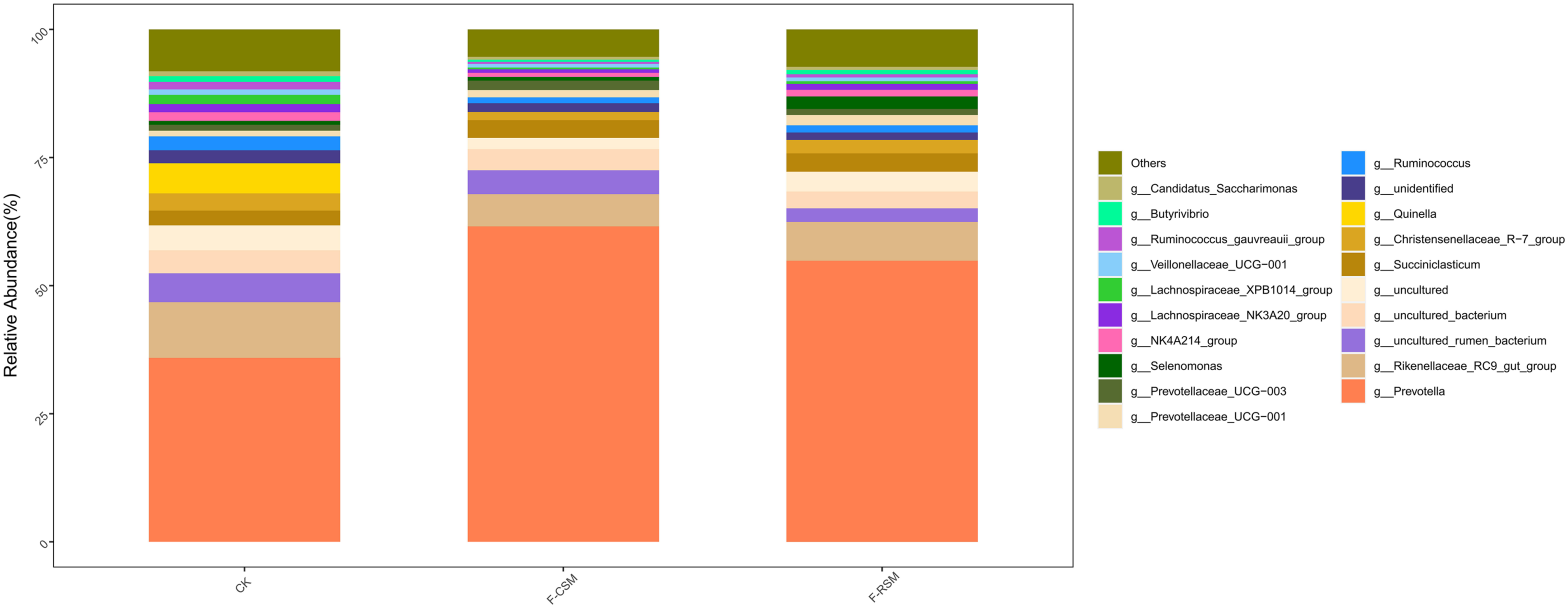
Effects of the diets on compositional profifiles of rumen microbiota of Hu sheep (Genus level; %); CK, non-fermented group; F-CSM, fermented Cotton seed meal TMR; F-RSM, fermented Rapeseed meal TMR.

**Figure 5:**
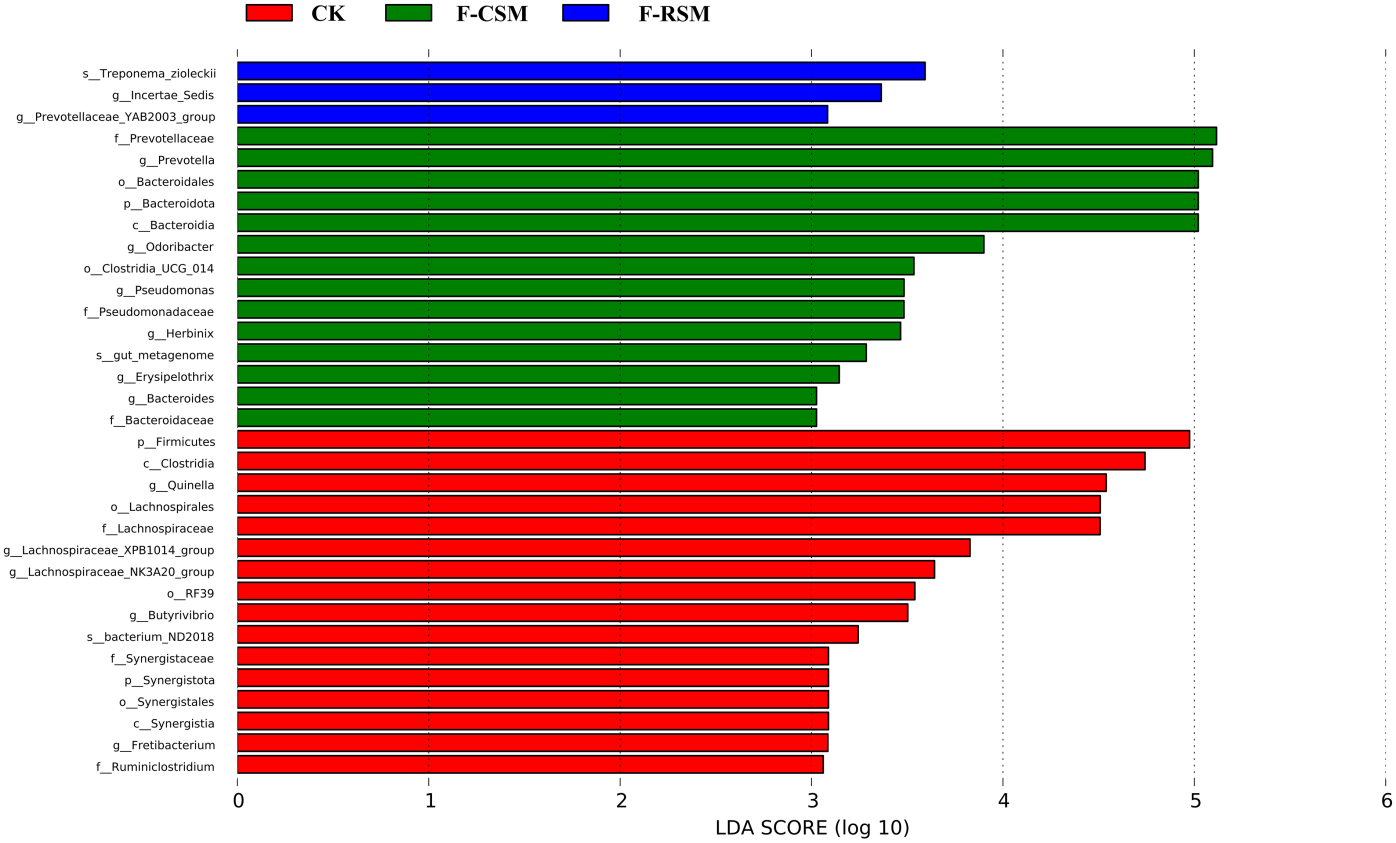
Effect of potential biomarkers was defined by LEfSe at Hu sheep rumen liquite (log_10_); CK, non-fermented group; F-CSM, fermented Cotton seed meal TMR; F-RSM, fermented Rapeseed meal TMR.

Seventeen bacterial phyla were found among all treatments, with *Bacteroidota*, *Firmicutes*, and *Proteobacteria* being the three most predominant. In comparison to that in the F-CSM group (58.72%), in the CK and F-RSM groups, the relative abundance of *Bacteroidetes* was greater (71.41 and 79.89%, respectively; *p* < 0.05). *Firmicutes* showed lower abundance in the CK group (18.30%) than in the F-CSM and F-RSM groups (*p* < 0.05). There were 195 diverse microbial genera found among all groups, but only 14 of them were determined to be the “major genera” since they each accounted for more than 0.5% of the total sequences through at least one treatment (Figure 3). Except for the genera *Prevotella* and *Christensenellaceae_R-7* (*p* < 0.05), no interaction (*p* > 0.05) was found for any bacterial abundance at the genus level. When compared to the CK group*, Prevotella* was more abundant in the F-CSM and F-RSM groups (*p* < 0.05), but the TMR type had no influence on their relative abundance (Figures 4, 5).

### 3.7. Effect on inferred functional pathways

In Table 5, the top 20 metabolic pathways are listed. The TMR type had a significant effect on 8 out of the 20 pathways. F-CSM and F-RSM exhibited higher (*p* < 0.05) relative abundance of the functions related to terpenoid, polyketide, lipid, and folding and transcription-associated metabolisms, despite the fact that there was no discernible difference between the two groups (*p* > 0.05).

**Table 5:** Effect of feeding different diets on predominant predicted gene pathways in the different ruminal ecological niches of growing Hu sheep.

Indices	Groups			SEM	*p* value
	CK^1^	F-CSM	F-RSM		
Carbohydrate metabolism	14.3	14.4	14.5	0.08	0.85
Metabolism of cofactors and vitamins	14.1	14.8	14.7	0.13	0.11
Amino acid metabolism	13.2	12.9	13.1	0.05	0.14
Metabolism of terpenoids and polyketides	8.9^a^	8.1^b^	8.3^b^	0.12	0.03
Metabolism of other amino acids	6.9	6.9	6.8	0.07	0.86
Replication and repair	6.3	6.4	6.4	0.03	0.67
Glycan biosynthesis and metabolism	5.7^b^	6.7^a^	6.3^a^	0.07	0.04
Energy metabolism	5.9	5.9	5.9	0.03	0.27
Lipid metabolism	4.2^a^	3.7^b^	3.9^b^	0.08	0.01
Translation	3.5	3.4	3.5	0.02	0.29
Folding, sorting and degradation	3.0^a^	2.8^b^	2.9^b^	0.03	0.01
Biosynthesis of other secondary metabolites	2.6	2.9	2.9	0.05	0.07
Nucleotide metabolism	2.2	2.2	2.2	0.02	0.17
Cell motility	2.0^a^	1.0^b^	1.5^ab^	0.15	0.02
Cell growth and death	1.7	1.8	1.8	0.14	0.08
Xenobiotics biodegradation and metabolism	1.5	2.1	1.7	0.19	0.24
Membrane transport	1.4^a^	1.3^b^	1.3^b^	0.03	0.05
Transcription	1.2^b^	1.3^a^	1.2^a^	0.01	0.05
Transport and catabolism	0.3	0.4	0.3	0.01	0.06
Signal transduction	0.3^a^	0.2^b^	0.2^b^	0.01	0.02

^a,b^Means with different superscripts within a same row differ (*p* < 0.05). ^1^CK, total mixed ration with soyabean meal; F-CSM, fermented total mixed ration with cottonseed meal; F-RSM, Fermented total mixed ration with rapeseed meal.

### 3.8. Relationship between VFA and rumen microbial diversity

**Figure 6:**
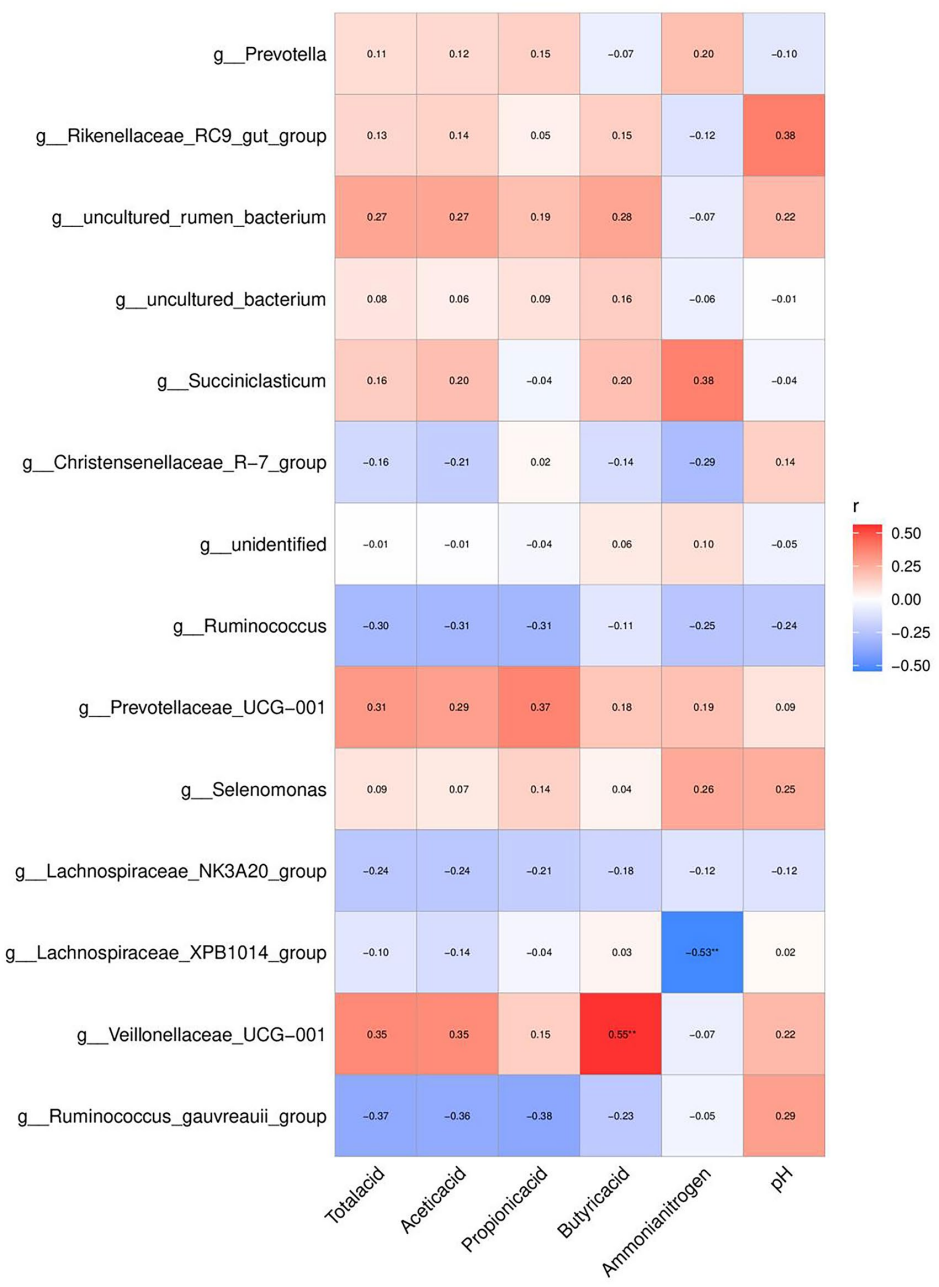
Effects of the diets on correlation between the top 20 relative abundances of genera, rumen VFA and enzyme activity mpositional profifiles of Hu sheep. *p < 0.05; **p < 0.01.

The relationship between the fermentation-related parameters and the relative abundance of the top 20 bacterial genera is shown in Figure 6. The relative abundances of *Veillonellaceae_UCG-001* and *Lachnospiraceae_XPB1014* correlated with rumen fermentation and NH_3_-N content (*p* < 0.05). While the relative abundance of *Lachnospiraceae_XPB1014* negatively correlated with NH_3_-N concentration (*r* = 0.53), the relative abundance of *Veillonellaceae_UCG-001* positively correlated with butyric acid content (*r* = 0.72).

## 4. Discussion

Due to the presence of anti-nutritional factors and the characteristics of the available substrates, diet composition has a significant impact on nutrient utilization and gastrointestinal physiology (Kiaie et al., 2014; Kim et al., 2017). Although it is generally recognized that TMR fermentation with microorganisms improves feed quality, there is an energy cost and a dry matter loss (Liang et al., 2017). Microbial fermentation can significantly reduce anti-nutritional factor content while improving palatability and nutritional value. CSM contains FG, while RSM contains glucosinolates, which affect the palatability and nutritional value of feed, reduce the production performance of animals, and also harm physical organs such as the liver and thyroid, greatly limiting its popularization and application in production. Hu et al. (2010) found that replacing SBM with 10% fermented CSM significantly increased the weight gain of sheep and reduced meat production costs. In a study by Xu et al. (2020), fermented feed partly breaks down the protein into the minor peptides & free amino acids, that imparts a pleasant acidic aroma to the feeds, enhances palatability as well as increases diet digestibility, which finally changes growth performance (Xu et al., 2020). However, our study showed that the F-CSM or F-RSM diet increased the overall body weight and daily weight gain in Hu sheep.

Rumen pH is the result of the interaction between VFAs in rumen digesta and buffer salts in saliva, absorption of VFAs by the rumen epithelium, and excretion of rumen digesta (Segata et al., 2011). It has an important impact on the rumen microbiota, rumen fermentation products, and rumen function, and is the most direct manifestation of rumen physiological conditions (Feng, 2004). Rumen pH is closely related to dietary composition and nutrient composition, and can comprehensively reflect the production, absorption, discharge, and neutralization of rumen microorganisms, metabolites, and organic acids (Nagaraja and Titgemeyer, 2007). The ruminal fluid pH varied from 5.93 to 6.39, which was within the normal range (Gilani et al., 2005), and among the groups, there was no significant different. These results indicate that various beneficial components contained in the fermented mixed meals were conducive to maintaining the stability of rumen pH, which could partially or completely replace SBM in the diet.

The VFAs produced during fermentation in the rumen are used as a source of energy in ruminants. VFAs produced by rumen fermentation, can provide up to 80% of the body’s energy and serve as the primary source of carbon for the proliferation of rumen microbes (Zhou et al., 2018). Acetic acid is the main precursor for milk fat synthesis, whereas propionic acid synthesizes the glucose required by the body through gluconeogenesis. As a result, propionic acid fermentation can provide the body more energy, which is beneficial for improving animal productivity and feed utilization (Spears et al., 2004). Studies have shown that microbial fermented feed can regulate rumen fermentation mode (Guo, 2016). Zhang Z. (2017) found that microbial fermented feed could significantly increase the contents of T-VFA, acetic acid and propionic acid and the ratio of acetate to propionate in the rumen of dairy cows through *in vitro* experiments, Guo (2016) also obtained similar results, and Chen et al. (2015) reported that fermented feed could increase the contents of rumen VFA, acetic acid and propionic acid. However, it had no significant effect on the ratio of acetate to propionate. Meanwhile, Li Z. et al. (2015) found that fermented feed could increase the content of rumen VFA of sheep and reduce the ratio of acetate to propionate. The present results show that rumen VFA concentration was the highest and the acetate/propionate ratio was the lowest in the animals fed with F-CSM. The F-CSM group showed lower VFA concentration than the F-RSM and CK groups. These results indicate that the substitution of fermented TMR contain CSM for SBM in Hu sheep diet is beneficial to the transformation of rumen fermentation type into propionic acid fermentation type and improve energy utilization efficiency.

The NH_3_-N concentration reflects the rate of decomposition of nitrogen-containing substances to produce NH_3_ and the uptake and utilization of NH_3_ by rumen microorganisms. A maximum concentration over 5 mg/dl can satisfy the needs of rumen microbial development. The NH_3_-N concentration in the rumen is essential for protein degradation and microbial protein synthesis (Li M. S. et al., 2015). In this experiment the replacement of SBM with F-CSM or F-RSM had a significant effect on rumen NH_3_-N content; however, the rumen NH_3_-N content was 7.09 mmol/l and 7.11 mmol/l, respectively, in the F-CSM and F-RSM groups, which is within the normal physiological range of rumen NH_3_-N (6–30 mg/d L; Yang et al., 2001). In our study, the diversity and richness of rumen bacteria did not significantly change when SBM was replaced with F-CSM or F-RSM in the diet of Hu sheep.

Rumen microorganisms decompose and utilize various secreted enzymes. Enzyme activity directly reflects the decomposition efficiency of feed nutrients in the rumen and determines the utilization efficiency of feed nutrients by animals. In this study, the activities of pepsin and cellulose in rumen fluid increased after replacing SBM with F-CSM. This may be because fermented TMR changes the pH of rumen fluid and provides favourable conditions for the reaction of digestive enzymes, thus improving the activity of digestive enzymes (Bowman and Asplund, 1988). In the F-RSM group, enzyme activity decreased. This could be the result of the anti-nutritional factors in RSM (Hassan et al., 2022b).

Researchers (Diao et al., 2013; Zhang et al., 2016; Zhou et al., 2017; Bi et al., 2018; Guo et al., 2021; Liu et al., 2021; Lu et al., 2021) have shown that the two dominant phyla in ruminants are *Bacteroidetes* and *Firmicutes*. The two most frequent taxa discovered in this study were *Bacteroidetes* and *Firmicutes*; the findings are consistent with other studies. In this study, we found that *Prevotella* and *Christensenellaceae R-7* were successively dominant at the genus level. Studies have shown that *Bacteroides* plays an important role in the degradation of non-fibrous substances, and *Firmicutes* mainly participate in the decomposition of fibrous substances (Evans et al., 2011), while non-fibrous substances produce propionic acid after rumen fermentation, while cellulose substances mainly produce acetic acid after degradation (Li et al., 2017). Our study the relative abundances of *Bacteroidetes* and *Firmicutes* in the F-CSM and F-RSM groups were greater than those in the CK group. These results indicate that fermented TMR can promote the growth and proliferation of *Bacteroidetes* in rumen, accelerate the degradation of non-fiber substances in diet, produce more propionic acid, provide more energy for the body, and promote the growth of animals.

The most abundant genus of Bacteroidetes is *Prevotella* (Zou et al., 2019). It participates in the metabolism of various microorganisms, has a high hemicellulose degradation ability, and can adapt to different dietary structures (Wolff et al., 2017). *Prevotella* is important in the degradation of crude protein, starch, xylan, and pectin (Li Z. et al., 2015; Jami et al., 2013). *Prevotella* relative abundance in the rumen was significantly greater in the F-CSM and F-RSM groups in this study. This suggests that F-CSM and F-RSM can replace SBM and help Hu sheep in improved nutrient absorption and digestion, thereby improving feed conversion rate. *Veillonellaceae* belongs to the phylum Firmicutes and can degrade and utilize cellulose (Long et al., 2022). We also found that the proportion of butyric acid was significantly positively correlated with the relative abundance of *Veillonellaceae UCG-001*. This indicated that *Veillonellaceae_UCG-001* was related to rumen VFA metabolism, which is consistent with the results of Thoetkiattikul et al. (2013). These results indicate that F-CSM and F-RSM can promote the growth of microorganisms, maintain intestinal structure and function, and regulate immunity in Hu sheep. *Lachnospiraceae_XPB1014*’s relative abundance and NH_3_-N content showed a negative correlation. These findings suggest a relationship between NH_3_-N concentrations and rumen bacteria in Hu sheep. Therefore, replacing SBM with F-CSM or F-RSM likely influenced the VFA and NH_3_-N contents, and ultimately impacted the rumen microbiota. Microbiota functional properties affect host-microbiome interaction (Lin et al., 2019; Zeng, 2020). The gene function prediction results showed that F-CSM and F-RSM can replace SBM in the diet of Hu sheep and have an important influence on their metabolism. Particularly, it promoted glycan biosynthesis and metabolism, indicating that F-CSM and F-RSM contributed to the maturation and stability of Hu sheep rumen microflora.

## 5. Conclusion

Substituting SBM with F-CSM or F-RSM in the diet of Hu sheep is beneficial for the conversion of microbiota from rumen fermentation type to propionic acid fermentation type and improving energy utilization efficiency. No negative effects were observed on rumen microbiota, and substituting SBM with F-CSM resulted in the best effect.

## Data availability statement

The original contributions presented in the study are included in the article/Supplementary material, further inquiries can be directed to the corresponding author.

## Ethics statement

The animal study was reviewed and approved by Committee of IFRCAAS (AEC-IFR-CAAS, Beijing, China). Written informed consent was obtained from the owners for the participation of their animals in this study.

## Author contributions

HR and HY: animal trial, data collection and evaluation, laboratory and statistical analysis, and writing. TM: critical manuscript review. QD: data evaluation and manuscript review. LxK and LyK: animal trial, laboratory analysis, and data collection. YT: study design, feed formulation, data evaluation, and critical manuscript review. All authors contributed to the article and approved the submitted version.

## Funding

This study was supported by the Innovation Project of Inner Mongolia (grant number: NMKJXM202110) and China Agriculture Research System of MOF and MARA (CARS-38).

## Conflict of interest

The authors declare that the research was conducted in the absence of any commercial or financial relationships that could be construed as a potential conflict of interest.

## Publisher’s note

All claims expressed in this article are solely those of the authors and do not necessarily represent those of their affiliated organizations, or those of the publisher, the editors and the reviewers. Any product that may be evaluated in this article, or claim that may be made by its manufacturer, is not guaranteed or endorsed by the publisher.

## Footnotes

^1^ http://galaxy.morganlangille.com
